# Biomarkers of aging and lung function in the normative aging study

**DOI:** 10.18632/aging.103363

**Published:** 2020-06-19

**Authors:** Cuicui Wang, Allan Just, Jonathan Heiss, Brent A. Coull, Lifang Hou, Yinan Zheng, David Sparrow, Pantel S. Vokonas, Andrea Baccarelli, Joel Schwartz

**Affiliations:** 1 Department of Environmental Health, Harvard T.H. Chan School of Public Health, Boston, MA 02115, USA; 2 Department of Environmental Medicine and Public Health, Icahn School of Medicine at Mount Sinai, New York, NY 10029, USA; 3 Department of Biostatistics, Harvard T.H. Chan School of Public Health, Boston, MA 02115, USA; 4 Department of Preventive Medicine, Feinberg School of Medicine, Northwestern University, Chicago, IL 60611, USA; 5 VA Normative Aging Study, VA Boston Healthcare System, Boston, MA 02130, USA; 6 Department of Medicine, Boston University School of Medicine, Boston, MA 02118, USA; 7 Department of Epidemiology and Environmental Health Sciences, Columbia University, New York, NY 10027, USA

**Keywords:** pulmonary health, DNA methylation, biological clock

## Abstract

Elderly individuals who are never smokers but have the same height and chronological age can have substantial differences in lung function. The underlying biological mechanisms are unclear. To evaluate the associations of different biomarkers of aging (BoA) and lung function, we performed a repeated-measures analysis in the Normative Aging Study using linear mixed-effect models. We generated GrimAgeAccel, PhenoAgeAccel, extrinsic and intrinsic epigenetic age acceleration using a publically available online calculator. We calculated Zhang’s DNAmRiskScore based on 10 CpGs. We measured telomere length (TL) and mitochondrial DNA copy number (mtDNA-CN) using quantitative real-time polymerase chain reaction. A pulmonary function test was performed measuring forced expiratory volume in 1 second / forced vital capacity (FEV_1_/FVC), FEV_1_, and maximum mid-expiratory flow (MMEF). Epigenetic-based BoA were associated with lower lung function. For example, a one-year increase in GrimAgeAccel was associated with a 13.64 mL [95% confidence interval (CI), 5.11 to 22.16] decline in FEV_1_; a 0.2 increase in Zhang’s DNAmRiskScore was associated with a 0.009 L/s (0.005 to 0.013) reduction in MMEF. No association was found between TL/mtDNA-CN and lung function. Overall, this paper shows that epigenetics might be a potential mechanism underlying pulmonary dysfunction in the elderly.

## INTRODUCTION

In 2050, there will be 84.7 million people ≥ 65 years of age in the U.S., almost double its estimated population of 43.1 million in 2012 [1]. The aging process reduces physiological capacity, which can result in functional impairment (e.g., lower lung function), chronic disease, and mortality. Chronological age is undoubtedly a major risk factor for aging-related diseases and death [2]. However, there is still great heterogeneity in the health outcomes of older individuals who have the same chronological age [3], especially for lung function, which has substantial heterogeneity among elderly individuals who are never smokers but have the same height and chronological age. These different vulnerabilities to age-related diseases and death are likely reflective of differences in their underlying biological aging processes [4]. The prevalence of pulmonary dysfunction increases sharply with age among elderly people. Moreover, poor lung function is related to impaired cognition [5, 6], adverse findings on brain imaging [7], and increased mortality [8–11]. However, the underlying mechanisms of lung function decline with aging are not fully characterized.

In the past decade, a number of biomarkers of aging (BoA) have emerged. For example, telomere length (TL) [12–14] and mitochondrial DNA copy number (mtDNA-CN) [15, 16] are well-known BoA and are related to aging-related diseases. As increasing body of evidence has suggested associations between aging and the epigenome [17], with DNA methylation (DNAm) levels being used to generate composite BoA [18–21]. The first generation of epigenetic BoA was calculated from DNAm levels alone, such as the Hannum epigenetic clock based on 71 5'-C-phosphate-G-3' sites (CpGs) in leukocytes [18], and the Horvath epigenetic clock based on 353 CpGs in multiple tissues [19]. Recently, the second generation of epigenetic BoA has been developed incorporating additional markers. For example, Levine et al. [4] created DNAm PhenoAge, based on 513 CpGs selected by predicting phenotypic age that were constructed using 9 clinical biomarkers (e.g., albumin). Lu et al. [20] generated DNAm GrimAge from 1,030 CpG sites that were highly predictable of 7 plasma proteins (e.g., DNA leptin) and smoking pack-years. Further, refined measures of BoA-epigenetic age acceleration were shown to be associated with age-related diseases and mortality [4, 20, 22]. The widely used BoA on epigenetic age accelerations include intrinsic and extrinsic epigenetic age acceleration (IEAA, EEAA) [22], PhenoAgeAccel [4], and GrimAgeAccel [20]. Additionally, Zhang’s DNAmRiskScore, which was calculated based on 10 CpG sites [21], was shown to be strongly associated with all-cause mortality [21]. The relationship between TL and lung function has been reported to be positive or null [23, 24]. Meanwhile, mtDNA-CN reduction is associated with chronic obstructive pulmonary disease [25]. Even epigenetic modifications have been shown to be associated with lung function in the elderly [26–28], however, no studies have investigated the associations between epigenetic aging biomarkers and lung function.

In this present study, we hypothesized that some of these BoA are associated with lower lung function. To examine this hypothesis, we tested for associations between each of seven BoA and three measures of lung function [forced expiratory volume in 1 second (FEV_1_), forced expiratory volume in 1 second / forced vital capacity (FEV_1_/FVC), maximum mid-expiratory flow (MMEF)] in the Normative Aging Study. Lower lung function in this paper is defined as a decrease in any of these three studied pulmonary function parameters.

## RESULTS

### Descriptive results

The present study included 696 elderly men with 1,070 visits during years of 1999-2013. Of the 696 subjects, 345 (50%) had two follow-up visits, and 29 (4%) completed three follow-up visits. Table 1 shows the personal characteristics of the participants across visits. The mean chronological age was 72.5, 75.2, and 76.3 in the first, second, and third visit, respectively. The cohort was well educated and made up of one third never smokers. Summary statistics of seven different BoA are presented in Table 2. The means of four age accelerations were close to zero, with GrimAgeAccel had the smallest standard deviation (4 years) (see Table 2).

**Table 1:** Personal characteristics of 696 (N=1,070) elderly white men from the Normative Aging Study, 1999-2013.

**Variable**	**First visit (N=696)**	**Second visit (N=345)**	**Third visit (N=29)**	**All visits (N=1,070)**
Chronological age (years)				
Mean ± SD	72.51 ± 6.74	75.20 ± 6.39	76.34 ± 5.94	73.48 ± 6.74
Range	55-100	57-94	64-86	55-100
BMI (kg/m^2^), mean ± SD	28.09 ± 4.10	27.81 ± 4.13	27.96 ± 4.32	28.00 ± 4.11
Height (m), mean ± SD	1.74 ± 0.07	1.74 ± 0.07	1.74 ± 0.07	1.74 ± 0.07
Smoking status, n (%)				
Never smokers	216 (31.03)	112 (32.46)	13 (44.83)	341 (31.87)
Current or former smokers	480 (68.97)	233 (67.54)	16 (55.17)	729 (68.13)
Pack-year smoked (years), mean ± SD	21.33 ± 25.33	19.07 ± 23.60	14.88 ± 20.18	20.43 ± 24.68
Years of education, mean ± SD	14.98 ± 2.97	15.17 ± 3.18	15.24 ± 3.45	15.05 ± 3.05
Corticosteroids, n (%)	51 (7.33)	30 (8.70)	1 (3.45)	82 (7.66)
Estimated cell types (%) [14]				
Monocytes	10.7 (1.7-25.1)	10.1 (4.0-18.7)	9.5 (6.4-12.6)	10.5 (1.7-25.1)
B cells	1.5 (0.0-31.4)	1.2 (0.0-17.5)	0.6 (0.0-5.3)	1.4 (0.0-31.4)
CD4^+^ T lymphocytes	8.7 (0.0-22.6)	8.3 (0.0-24.1)	8.5 (0.9-20.1)	8.6 (0.0-24.1)
CD8^+^ T lymphocytes	8.7 (0.0-20.2)	8.5 (0.0-15.5)	8.0 (3.0-13.8)	8.6 (0.0-20.2)
Natural killer cells	7.3 (0.0-22.1)	7.5 (0.0-25.6)	7.8 (1.6-21.6)	7.4 (0.0-25.6)
FEV_1_ (mL 1^st^ sec), mean ± SD	2500 ± 580	2540 ± 610	2680 ± 440	2520 ± 586
FEV_1_/FVC, mean ± SD	0.75 ± 0.08	0.74 ± 0.07	0.73 ± 0.08	0.75 ± 0.08
MMEF (L/s), mean ± SD	0.24 ± 0.11	0.23 ± 0.10	0.22 ± 0.08	0.24 ± 0.11

Abbreviations: SD = standard deviation; BMI = body mass index; FEV_1_ = forced expiratory volume in 1 second; FVC = forced vital capacity; MMEF = maximum mid-expiratory flow.

**Table 2:** Summary statistics of different biomarkers of aging in 1,070 visits among 696 elderly white men from the Normative Aging Study, 1999-2013.

**Variables**	**mean ± SD**	**Percentile**		
		**5th**	**50th**	**95th**
Epigenetic BoA				
GrimAgeAccel (years)	0.01 ± 4.07	-5.52	-0.63	7.82
PhenoAgeAccel (years)	-0.17 ± 5.99	-9.31	-0.56	10.09
IEAA (years)	-0.16 ± 5.08	-7.87	-0.62	8.26
EEAA (years)	-0.10 ± 5.81	-9.41	-0.24	9.61
Zhang’s DNAmRiskScore	-1.87 ± 0.44	-2.54	-1.90	-1.07
Non-epigenetic BoA				
TL	1.25 ± 0.49	0.58	1.19	2.15
mtDNA-CN	1.03 ± 0.22	0.73	1.01	1.38

Abbreviations: SD = standard deviation; IEAA = intrinsic epigenetic age acceleration; EEAA = extrinsic epigenetic age acceleration; TL = Telomere length; mtDNA-CN = mitochondrial DNA copy number.

The trends of lung function and methylation based BoA among 29 men who had three visits are presented in supplementary material (Supplementary Figures 1 and 2). The supplementary material also contains trends of four DNAm ages (i.e. DNAm GrimAge, DNAm PhenoAge, the Horvath’s Clock, and the Hannum’s Clock), which increased consistently across visits (Supplementary Figure 3). We also generated four DNAm ages (i.e., DNAm GrimAge, DNAm PhenoAge, the Horvath’s clock, and the Hannum’s clock) on the Age Calculator website. Summary statistics of chronological age and four epigenetic ages are presented in Supplementary Table 1. Supplementary Figure 4 shows the distribution of chronological age and four DNAm ages, among which the distribution of DNAm GrimAge was closest to the one of chronological age (Supplementary Figure 4).

**Figure 1:**
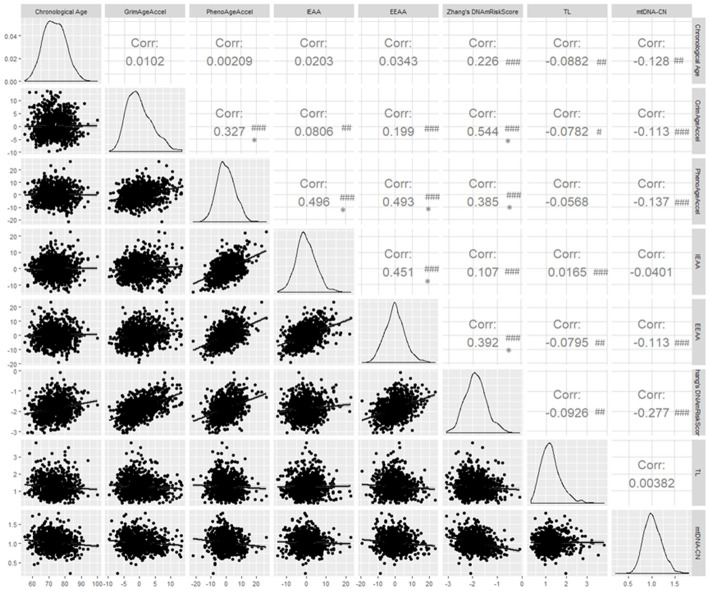
**Pairwise correlations between chronological age and seven biomarkers of aging.** # *P* ≤ 0.05; ## *P* ≤ 0.01; ### *P* ≤ 0.001; * Clinically significant; Corr = correlation coefficient.

We calculated pairwise correlations between chronological age and the seven BoA (Figure 1). According to Hung et al. [29], only correlation coefficient (r) 0.30 should be considered as "clinically significant" when *P* < 0.05. In this study, chronological age was not clinically associated with seven BoAs, while it was statistically significantly correlated with Zhang’s DNAmRiskScore (correlation coefficient ® = 0.23; *P* ≤ 0.001), TL (r = -0.09; *P* ≤ 0.01), and mtDNA-CN (r = -0.13; *P* ≤ 0.01). GrimAgeAccel was clinically associated with PhenoAgeAccel (r = 0.33; *P* ≤ 0.001) and Zhang’s DNAmRiskScore (r = 0.54; *P* ≤ 0.001). IEAA and EEAA were also highly correlated with each other (r = 0.45; *P* ≤ 0.001). TL and MtDNA-CN was not clinically associated with other BoAs. We also computed pairwise correlations between chronological age and four types of epigenetic ages. All of them were highly correlated with each other (Supplementary Figure 5). For epidemiologic purposes, non-clinically significant associations may still represent important associations.

### BoA and lung function

**Figure 2:**
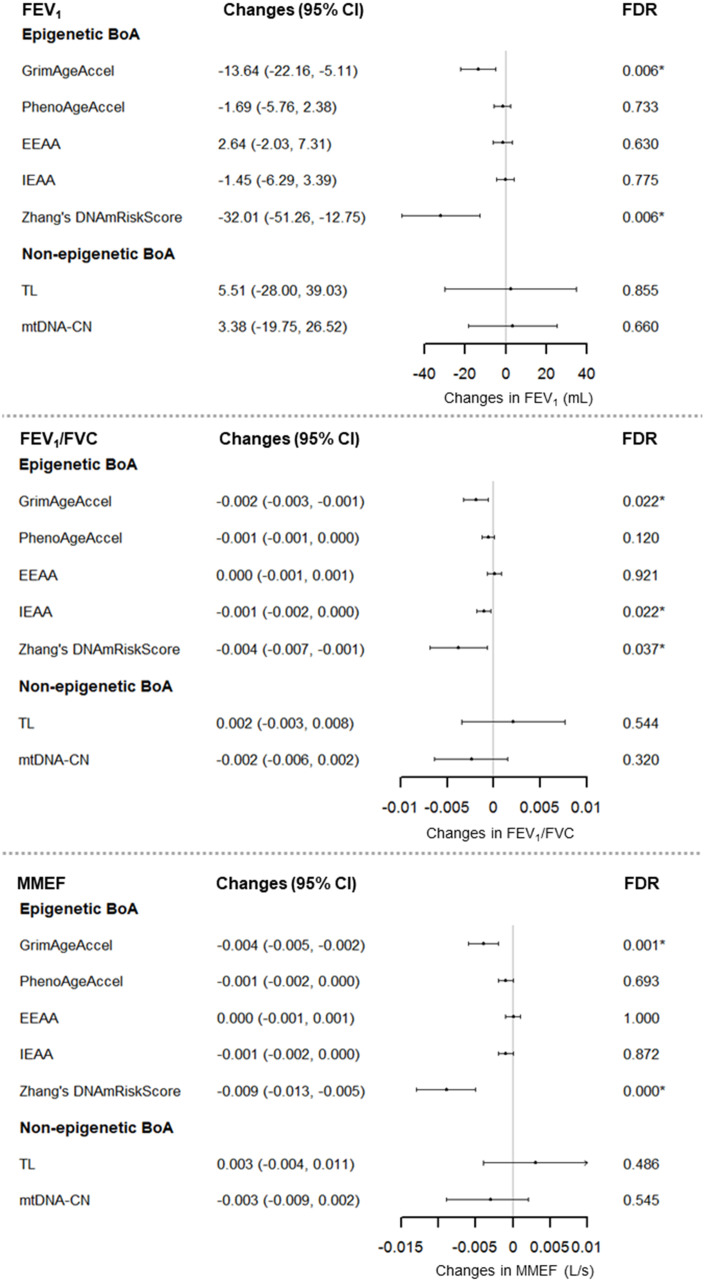
**Associations between Seven BoAs and lung function for 696 men (1,070 visits), the NAS, 1999-2013.** For GrimAgeAccel, PhenoAgeAccel, TL, and mtDNA-CN, we adjusted for chronological age, BMI, height, smoking status, cigarette pack-years, years of education, corticosteroid use, estimated cell types, and batch effects. For EEAA and IEAA, we adjusted for chronological age, BMI, height, smoking status, cigarette pack-years, years of education, corticosteroid use, and batch effects. For Zhang’s DNAmRiskScore, we adjusted for chronological age, BMI, height, smoking status, cigarette pack-years, years of education, corticosteroid use, estimated cell types, batch effects, and technical covariates: Non polymorphic Red, Specificity I Red, Bisulfite Conversion I Red, Bisulfite Conversion II, Extension Red. Abbreviations: IEAA = intrinsic epigenetic age acceleration; EEAA = extrinsic epigenetic age acceleration; TL = Telomere length; mtDNA-CN = mitochondrial DNA copy number; BoA = biomarkers of aging; BMI = body mass index; FDR_B-H_ = Benjamin-Hochberg false discovery rate.

Figure 2 shows the results of the primary analyses. FEV_1_, FEV_1_/FVC and MMEF was significantly negatively associated with each of GrimAgeAccel and Zhang’s DNAmRiskScore. For example, each increment of one year in GrimAgeAccel was associated with a decrease in FEV_1_ of 13.64 mL (95% CI: -22.16 to -5.11; FDR_B-H_ = 0.006), in FEV_1_/FVC of 0.002 (95% CI: -0.003 to -0.001; FDR_B-H_ = 0.022), and in MMEF of 0.004 L/s (95% CI: -0.005 to -0.002; FDR_B-H_ < 0.001). In addition, a 0.2 increase in Zhang’s DNAmRiskScore was associated with a decrease in FEV_1_ of 32.01 mL (95% CI: -51.26 to -12.75; FDR_B-H_ = 0.006), in FEV_1_/FVC of 0.004 (95% CI: -0.007 to -0.001; FDR_B-H_ = 0.037), and in MMEF of 0.009 L/s (95% CI: -0.013 to -0.005; FDR_B-H_ < 0.001). IEAA was related to lower FEV_1_/FVC. No association was found between lung function and non-epigenetic BoA.

### Sensitivity analyses

Sensitivity analyses further validated the reliability of our primary findings. Results were remarkably consistent after excluding visits at which corticosteroid use was reported (N=82) (Supplementary Figure 6) or adjusting for coronary heart disease (31% of visits), stroke (8%), and diabetes (15%) (Supplementary Figure 7). When we used inverse probability weighting to account for potential selection bias, the associations were similar to those from the primary analysis (Supplementary Figure 8). Subset analyses suggested strong negative associations of lung function and BoA (e.g., GrimAgeAccel, and Zhang’s DNAmRiskScore) among smokers, whereas almost no associations between BoA and lung function among never smokers were present (Supplementary Figure 9A and 9B).

## DISCUSSION

In this longitudinal cohort of 696 elderly males, we found that GrimAgeAccel and Zhang’s DNAmRiskScore were associated with lower lung function, including FEV_1_, FEV_1_/FVC, and MMEF. These effects were stable in three sensitivity analyses. These results suggest that epigenomic variation might shed new insights into the pathogenesis of lower lung function with age. To the best of our knowledge, this is the first study to examine the associations between epigenetic BoA and lung function. Non-epigenetic aging biomarkers, including TL and mtDNA-CN were not related to pulmonary function.

According to the American Lung Association, the lung matures by age 20-25 years, and its function declines gradually after the age of 35 [30]. Poor lung function is often indicative of risk of diseases [5–7] and mortality [8–11], and hence a public health priority. In this study, we focused on three spirometric indices that provide slightly different information on lung function. FEV_1_ is a measure of overall lung function. FEV_1_/FVC often decreases due to changes in elasticity, airspace enlargement, and other physiologic change that occur with age [31]. In this study, we found that mean FEV_1_/FVC was high at 0.75 in the first visit and decreased slightly in the next two visits, which was consistent with our previous study [26]. The potential reasons were: 1). the subjects in NAS were free of known chronic medical conditions at enrollment; 2). they have high educational attainment and relative healthy lifestyle. MMEF, expressed as forced expiratory flow between 25-75% of FVC, is determined by the size of the mid-airways. A low flow of rate of MMEF reflects airway narrowing or obstruction. In the present study, TL and mtDNA-CN were not related to lung function, whereas GrimAgeAccel and Zhang’s DNAmRiskScore were significantly and stably associated with lower lung function, including FEV_1_, FEV_1_/FVC, and MMEF. IEAA was significantly related to lower FEV_1_/FVC.

In keeping with the unprecedented growth rate of the world’s aging population, there is a clear need to better understand the biological aging process. TL shortens with each cell division [32], and thus serves as a measure of biological aging. Although a number of studies showed TL was associated with several aging-related diseases [14, 15], we didn’t find the relationship between TL and lower lung function in the main analyses. Consistent with our finding, Andujar et al. [33] found no association between TL at baseline and FEV_1_ decline among 448 middle-aged adults after 11 years of follow-up in Europe. It could be because telomere attrition does not have marked effects on cell physiology until a critical TL is reached [32], at which point the cell becomes senescent [34], or this result may be specific to lung function.

Mitochondria are double-membrane-bound organelles, present in almost all mammalian cells. Although most of a cell’s DNA is contained in the cell nucleus, the mitochondrion has its own independent genome, which is more susceptible to oxidative attack due to lack of introns and protective histone proteins as well as limited capacity to repair [35]. The most important roles of mitochondria are the generation of adenosine triphosphate and regulation of oxidative stress [36]. Decrease in mtDNA-CN, which serves as a surrogate marker of mitochondrial function, has been linked with diseases [15, 16]. However, there are differences among tissues in mitochondrial number, function, protein composition [37], which might partly explain why the present study did not find an association between leucocyte mtDNA-CN and lung function.

Several studies have suggested that epigenetic mechanisms like DNAm may provide explanation for lower lung function [26–28]. For example, Carmona et al. [28] found a positive association between DNAm of *aryl-hydrocarbon receptor repressor* (*AHRR*) gene and FEV_1_, and MMEF in the NAS and the Cooperative Health Research in the Region of Augsburg cohort. Lepeule et al. [26] showed that decreased methylation of genes *carnitine O-acetyltransferase*, *coagulation factor-3*, and *toll-like receptor 2* was associated with lower lung function in the NAS.

Over the last six years, several epigenetic BoA have been shown associated with health [35, 36, 38, 39]. The first generation of epigenetic BoA was developed to predict chronological age (correlation coefficients > 0.9), such as the Hannum’s clock, which was based on 71 CpG sites from adults’ blood DNA [18]. Instead of a single-tissue DNAm aging biomarker, Horvath et al. [19] created the Horvath’s clock based on 353 CpGs using over 30 different tissue and cell types collected from children and adults. Nevertheless, there are two main limitations of the first generation of BoA. First, while they exhibit statistically significant associations with many age-related diseases [38, 39], the effect sizes are typically small or moderate [4]. Second, only weak associations with clinical measures of physiological dysregulation were observed [35, 36].

In the interest of obtaining more powerful DNAm-based estimators of epigenetic BoA, Levine et al. [4] developed DNAm PhenoAge based on 513 CpGs, which derived from clinical biomarkers. While the first generation selects CpGs to optimize prediction of chronological age, the CpGs in DNAm PhenoAge is optimized to predict a multi-system proxy of physiological dysregulation (i.e., phenotypic aging). In doing so, the investigators were able to not only capture CpGs that exhibited strong correlations with chronological age, but those that capture variations in the risk of death and diseases among same aged individuals [4].

Recently, Lu et al. [20] employed a novel two-stage procedures and calculated DNAm GrimAge, which is a linear combination of chronological age, sex, DNAm-based surrogate biomarkers, and smoking pack-years. Compared with DNAm PhenoAge, DNAm GrimAge includes more clinical biomarkers, such as cardiovascular disease related plasma proteins (e.g. C-reactive protein, and growth differentiation factor-15). Hence, DNAm GrimAge outperforms all other DNAm-based biomarkers, on a variety of health-related metrics [20].

Since chronological age was highly correlated with epigenetic ages (Supplementary Figure 5 in the supplementary material), we used the corresponding age accelerations, which are new indices of accelerated biological aging and display a high risk of various health conditions (e.g., lung cancer, all-cause mortality, Parkinson’s disease, and Alzheimer’s disease) [22, 36, 39, 40] to investigate the relationship between epigenetic BoA and lung function in this study. IEAA is defined as the residual resulting from regressing the Horvath’s clock on chronological age and blood cell count estimates. By definition, IEAA does not depend on chronological age or on blood cell counts [22]. EEAA is calculated in three steps: i) calculation of the Hannum’s clock; ii) inclusion the blood cell types to generate the age estimate (called BioAge4 in epigenetic clock software); iii) calculation the residual from a univariate model regressing BioAge4 on chronological age [22]. EEAA captures age-related changes in blood cell types. Moreover, IEAA and EEAA, by definition, are not correlated (r<0.01) with chronological age.

GrimAgeAccel is the corresponding raw residual in the linear regression model with DNAm GrimAge regressed on chronological age (i.e. the difference between the observed value of DNAm GrimAge minus its expected value) [20]. Similarly, PhenoAgeAccel is the residual calculated by a linear regression model in which DNAm PhenoAge is the outcome, and chronological age is the independent variable [4]. A positive value of GrimAgeAccel/PhenoAgeAccel indicates that the biological age of the observation is higher than expected based on chronological age. GrimAgeAccel and PhenoAgeAccel, which by definition, are not correlated (r<0.01) with chronological age. To date, only limited studies have investigated the relationship between epigenetic age acceleration and pulmonary health. For instance, Levine et al. [36] found that IEAA was significantly associated with lung cancer incidence (hazard ratio: 1.5, *P* = 0.003) among 2,029 females in the Women’s Health Initiative.

Unlike the above epigenetic BoA, which are in units of years, Zhang’s DNAmRiskScore is a continuous risk score ranging from -4 to 0. Zhang et al. [21] developed this risk score based on ten CpG sites. The DNAmRiskScore is a predictor of all-cause mortality. Even though it includes only 10 CpGs, Zhang’s DNAmRiskScore had significantly and stable negative associations with lung function in our analyses. To understand this, we investigated the common CpG sites among different epigenetic BoA. Since DNAm GrimAge has been patented, Liu et al. could not release the 1,030 CpG identifiers. We found that DNAm PhonoAge had 41 CpGs in common with the Horvath’s clock, and 6 CpGs in common with the Hannum’s clock. The Horvath’s clock shared 6 CpG sites with the Hannum’s clock. However, Zhang’s DNAmRiskScore didn’t share any CpGs with the other three BoA [4, 18, 19, 21].

To further explore Zhang’s DNAmRiskScore, we investigated the genes where these CpG sites are located. Interestingly, one of the 10 CpGs (cg05575921) maps to the *AHRR* gene. Recently, studies have shown that hypo-methylation in *AHRR* at cg05575921 not only strongly reflects smoking history, but relates to lower lung function [41], Additionally, it predicts future smoking-related mortality [42]. Another CpG site (cg19572487) maps to the *retinoic acid receptor alpha* gene, which plays an important role in various human cancers such as lung cancer [35, 39]. Moreover, both cg05575921 and cg19572487 are not contained in the other three epigenetic BoA in the present study (We were not able to investigate the 1,030 CpGs involved in DNAm GrimAge due to the patent issue as mentioned above).

For smoking-stratified analysis, some epigenetic BoA were associated with lower lung function in ever smokers (current and former). The subgroup analyses indicate that the significant associations between lower lung function and BoA were evident only among smokers. With aging, lung function experiences a progressive decrease due to changes in physiology and structure in the lung. In the present study, among never smokers, the lung function decline was mainly associated with chronological age, BMI, and height; whereas among smokers, the impaired pulmonary function was related to pack-years and epigenetic BoA. These findings indicate that smoking status may result in significant abnormal alteration in age-related genes. Epigenomic variation may help to explain features of lower lung function and related pathophysiology in smokers. Additionally, the findings support the importance of smoking cessation, especially for elderly people.

Several study limitations should be noted. First, our findings are based on an elderly cohort of white males. Hence, additional studies involving diverse demographic groups (younger or women) will be helpful to complement our conclusions. Second, we used an old spirometer to measure pulmonary health (it was not replaced to ensure continuity of the measurements). However, the acceptability of spirograms was judged according to American Thoracic Society/European Respiratory Society criteria [43] and the spirometric values were adjusted by body temperature and pressure [44]. Third, we cannot rule out unmeasured confounding. However, we used existing literature and a *priori* knowledge of clinical relevance to adjust for potential confounders.

The major strengths in the study are the following: First, we investigated different types of BoA, including non-epigenetic- and epigenetic-based measures. Therefore, our work is based on multifaceted analyses of the associations between age estimators and lung function. Second, this evidence not only sheds new light into pathogenesis and susceptibility to lower lung function, but since DNAm changes are reversible, epigenetic BoA might be useful for identifying anti-aging interventions in the lung, especially for elderly smokers.

In conclusion, DNAm GrimAge, and Zhang’s DNAmRiskScore were associated with lower lung function. TL and mtDNA-CN were not related to pulmonary impairment. Epigenetic mechanisms such as DNAm may provide further explanation for decreases in lung function as individual age.

## MATERIALS AND METHODS

### Study population

The Normative Aging Study is a closed cohort established by the U.S. Veterans Administration in 1963 [45]. It enrolled 2,280 men volunteers living in the Boston area, aged between 21-80 years, who were free of known chronic medical conditions at enrollment. The study population had undergone clinical examinations every 3 to 5 years. These examinations took place in the morning after an overnight fast. Data on demography, health status, mediation use, and blood sample were collected at each visit.

**Figure 3:**
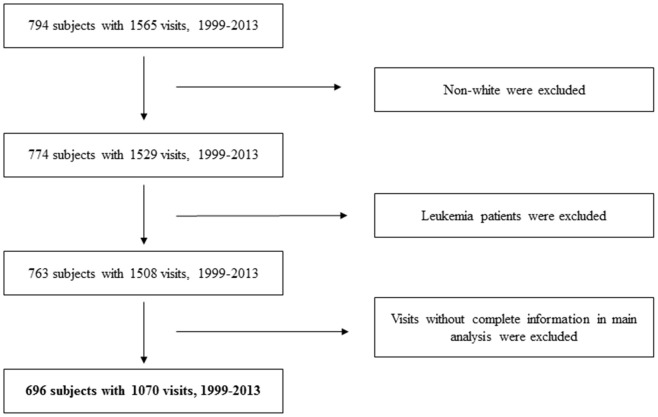
**Flowchart of study participants.**

We narrowed down our analyses to visits with DNA samples, which were collected from 1999 to 2013 [46]. To reduce study heterogeneity, those who were non-white (3%) were excluded. Subjects with a diagnosis of leukemia (n=11) were also be removed, due to its potential influence on DNAm [19]. This study was approved by the Institutional Review boards of the Department of Veterans Affairs and the Harvard TH Chan School of Public Health. All participants provided their written informed consent. The flowchart of our study participants is shown in Figure 3.

### Pulmonary health measures

Spirometry was assessed in the standing position with a nose clip using a Collins Survey Spirometer with Eagle II Microprocessor (Warren E. Collins, Braintree, Massachusetts), and a protocol that adhere to American Thoracic Society standards [44]. Acceptability of spirograms was judged according to American Thoracic Society/European Respiratory Society criteria [43]. A minimum of three acceptable spirograms was obtained, of which at least two were reproducible within 5% for FVC, and FEV_1_. Spirometric values were adjusted by body temperature and pressure [44].

### BoA measures

### Epigenetic BoA

DNA samples were extracted from the buffy coat of whole blood collected from each visit using the QIAamp DNA Blood Kit (Qiagen, CA, USA) between 1999 and 2013. To ensure a similar age distribution and avoid confounding across plates, we randomized samples based on a two-stage age stratified algorithm [47]. We measured DNAm using the Illumina Infinium HumanMethylation450 BeadChip (Infinium HD Methylation protocol, Illumina, San Diego, CA, USA), which includes ~ 485, 000 CpGs at a single nucleotide resolution. In the quality control step, we removed samples with a detection p-value < 0.01 based on an estimation of the background distribution using non-specific fluorescence [48], and corrected for dye-bias [49].

In the website of New Methylation Age Calculator (https://dnamage.genetics.ucla.edu/new), we uploaded our methylation data file and sample annotation file. We selected “Normalize Data”, and “Advanced Analysis” before submitting our data. We acquired four measures of BoA - epigenetic age accelerations (i.e. GrimAgeAccel, PhenoAgeAccel, EEAA, IEAA), and four DNAm ages (i.e. DNAm GrimAge, DNAm PhenoAge, the Hannum’s clock, the Horvath’s clock) via email, which was used to register in the website.

Zhang’s DNAm RiskScore was calculated based on 10 selected CpGs. The formula [21] is: cg01612140*(-0.38253) + cg05575921*(-0.92224) + cg06126421*(-1.70129) + cg08362785*(2.71749) + cg10321156*(-0.02073) + cg14975410*(-0.04156) + cg19572487*(-0.28069) + cg23665802*(-0.89440) + cg24704287*(-2.98637) + cg25983901*(-1.80325).

### *Non-epigenetic BoA*

We measured leukocyte TL by quantitative real-time polymerase chain reaction [50]. Relative leukocyte TL was calculated using a method previously described [12, 13]. Briefly, it is calculated as the ratio of the telomere (T) repeat copy number to a single-copy gene (S) copy number (T:S ratio) in a given sample, and reported as relative units expressing the ratio between TL in the test DNA and TL in a DNA pool used to generate a stand curve in each PCR run. We ran all samples in triplicates, and the average of the three T measurements was divided by the average of the three S measurements to calculate the average T:S ratio.

For mitochondrial DNA copy number (mtDNA-CN), we also adapted a real-time polymerase chain reaction and quantified it using mtDNA 12S ribosomal RNA TaqMan probe, as described in detail previously [51]. We adapted a multiplex RT-PCR to measure mtDNA content [52]. The mtDNA 12 S ribosomal ribonucleic acid TaqMan (Applied Biosystems, Waltham, Massachusetts) probe (6FAM-5′ TGCCAGCCACCGCG 3′-MGB) was used to measure mtDNA-CN. The sequences of primers used for amplification in mtDNA were mtF805 (5′CCACGGGAAACAGCAGTGATT3′) and mtR927 (5′CTATTGACTTGGGTTAATCGTGTGA3′). All samples were run in triplicate. The mean of the three measurements was used for statistical analyses.

### Statistical analysis

Separate linear mixed-effect models were applied to estimate the associations between each BoA and lung function, with subject-specific intercept to account for repeated estimation of lung function. For outcome Y _*ij*_ representing either FEV_1_, FEV_1_/FVC or MMEF for subject i on occasion j, the model is

$${\text{Y}}_{ij}=\beta_{0}+\mu_{i}+\beta_{1}{\text{BoA}}_{ij}+\beta_{2}{\text{X}}_{2ij}+\cdots +\beta_{p}X_{pij}+\varepsilon_{ij},$$ (1)

where β_0_ is the intercept, μ_i_ is the random intercept for participant i; β_1_ is the association between a chosen BoA measure and lung function for that subject on that occasion; X_2ij_ to X_pij_ represent the p-1 covariates, the selection of which differed slightly depending on the measure of BoA used in the analysis (see below). Effect estimates are expressed per one year increase in GrimAgeAccel / PhenoAgeAccel / IEAA / EEAA, or 0.2 increase in Zhang DNAmRiskScore / mtDNA-CN, or 1 increase in TL.

We selected the fundamental covariates *a priori*: chronological age, body mass index (BMI), height, smoking status [never smokers who never smoked before versus former or current smokers)], cigarette pack-years, years of education, corticosteroid use, estimated cell types (CD4^+^ T lymphocytes, CD8^+^ T lymphocytes, natural killer cells, B cells, and monocytes) derived by the method of Houseman et al. [53], batch effects for all BoA except for IEAA which measures “pure” epigenetic aging effects that are not confounded by differences in blood cell counts, and EEAA which is accounted for cell types in the calculation steps [22].

For Zhang’s DNAmRiskScore, which was calculated from the formula (1), we additionally adjusted for important technical covariates because normalization on the methylation data might diminish biologic signals. Therefore, we included important technical covariates from the control metrics monitoring the performance of various experimental steps [54]. The potential technical covariates included time when DNA methylation measures were performed and experimental parameters including Staining Green, Staining Red, Extension Green, Extension Red, Hybridization High Medium, Hybridization Medium Low, Target Removal 1, Target Removal 2, Bisulfite Conversion I Green, Bisulfite Conversion I Red, Bisulfite Conversion II, Specificity I Green, Specificity I Red, Specificity II, Non Polymorphic Green, and Non Polymorphic Red. We used elastic-net regularized generalized linear model and selected five important technical covariates: Non polymorphic Red, Specificity I Red, Bisulfite Conversion I Red, Bisulfite Conversion II, Extension Red.

Statistical analysis were performed using R (3.5.1) with “lme4” package (linear mixed-effect model) and “glmnet” package (lasso and elastic-net regularized generalized linear model). To adjust for multiple comparisons, we applied Benjamin-Hochberg false discovery rate (FDR_B-H_) and set the false positive threshold as 0.05 [55].

### Sensitivity analyses

We repeated the models in several different ways to conduct sensitivity analyses. First, we excluded visits at which corticosteroid use was reported (N=82); second, we adjusted models for chronic diseases (coronary heart diseases, stroke, and diabetes) when their DNA samples were collected; third, to adjust for the possibility that healthier men are more likely to return for subsequent exams, we applied inverse probability weighting [12] using logistic regression to calculate the probability of having a subsequent visit given chronological age, education, BMI, blood pressure, smoking status, cigarette pack years, alcohol consumption, C-reactive protein, asthma, chronic bronchitis, and emphysema at previous visit. Finally, we divided the subjects into two groups based on their smoking status, and tested the BoA-lung function associations separately. One groups were subjects who never smoked before (never smokers), and the other one were subjects who were former or current smokers.

## Supplementary Material

Supplementary Figures

Supplementary Table
