# Supplementary material for: Biomarkers of aging and lung function in the normative aging study
Source: Aging (Albany NY). 2020 Jun 19;12(12):11942–66. doi: 10.18632/aging.103363 (PMC7343502; doi:10.18632/aging.103363)
Supplement: Supplementary Figures [file aging-12-103363-s002..pdf]

SUPPLEMENTARY FIGURES

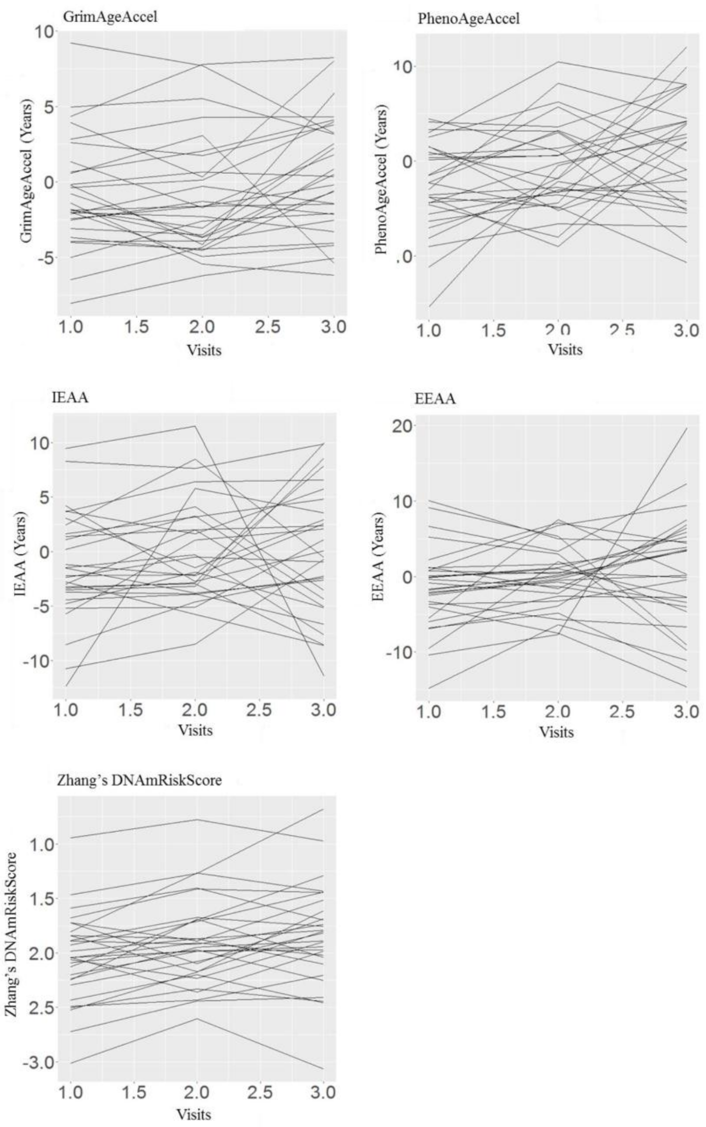

Supplementary Figure 1. The trend of methylation based BoAs over three visits among 29 men.

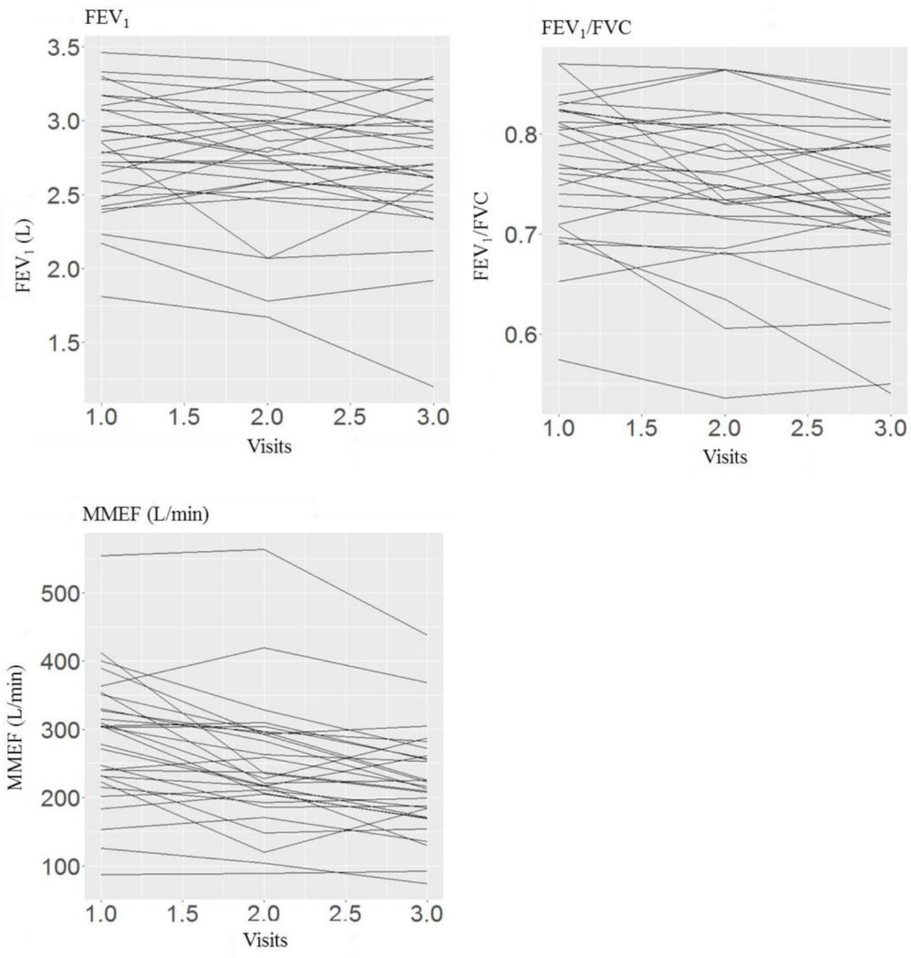

Supplementary Figure 2. The trend of lung function over three visits among 29 men.

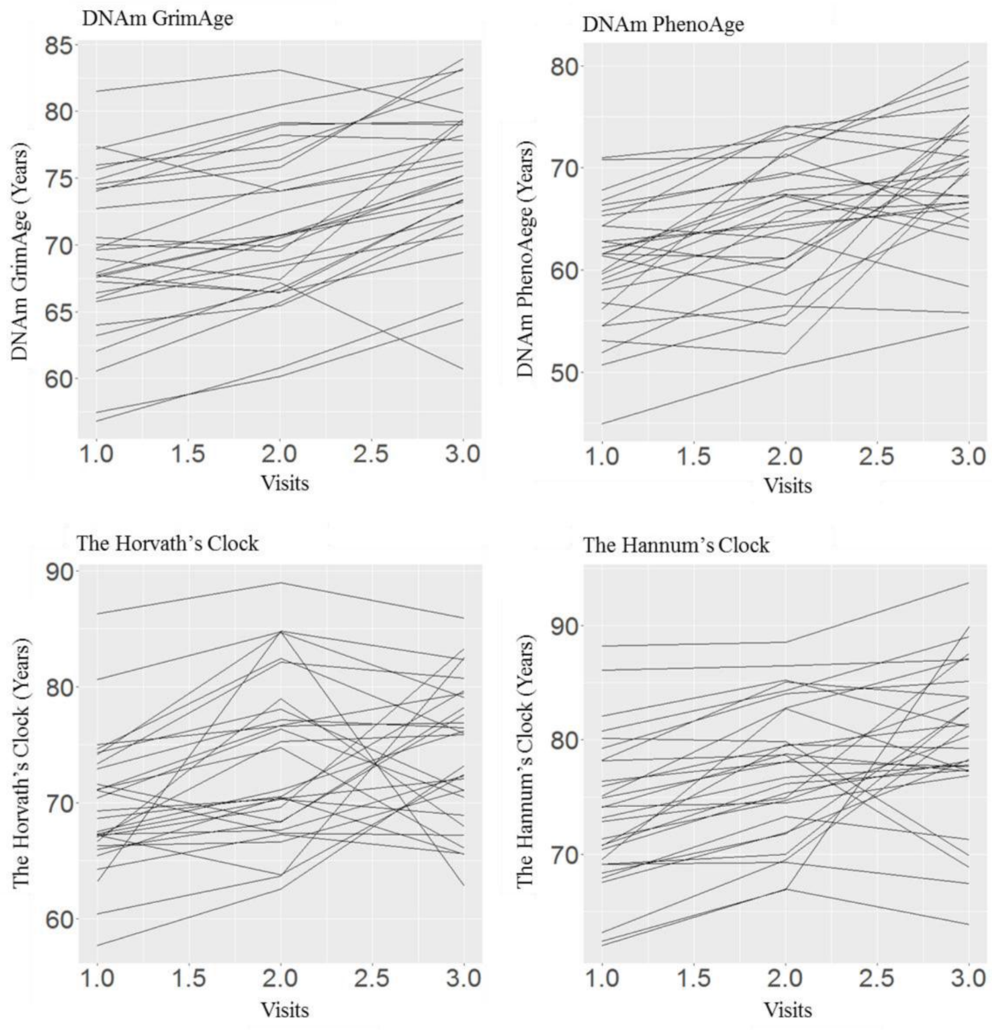

Supplementary Figure 3. The trend of four biological ages over three visits among 29 men.

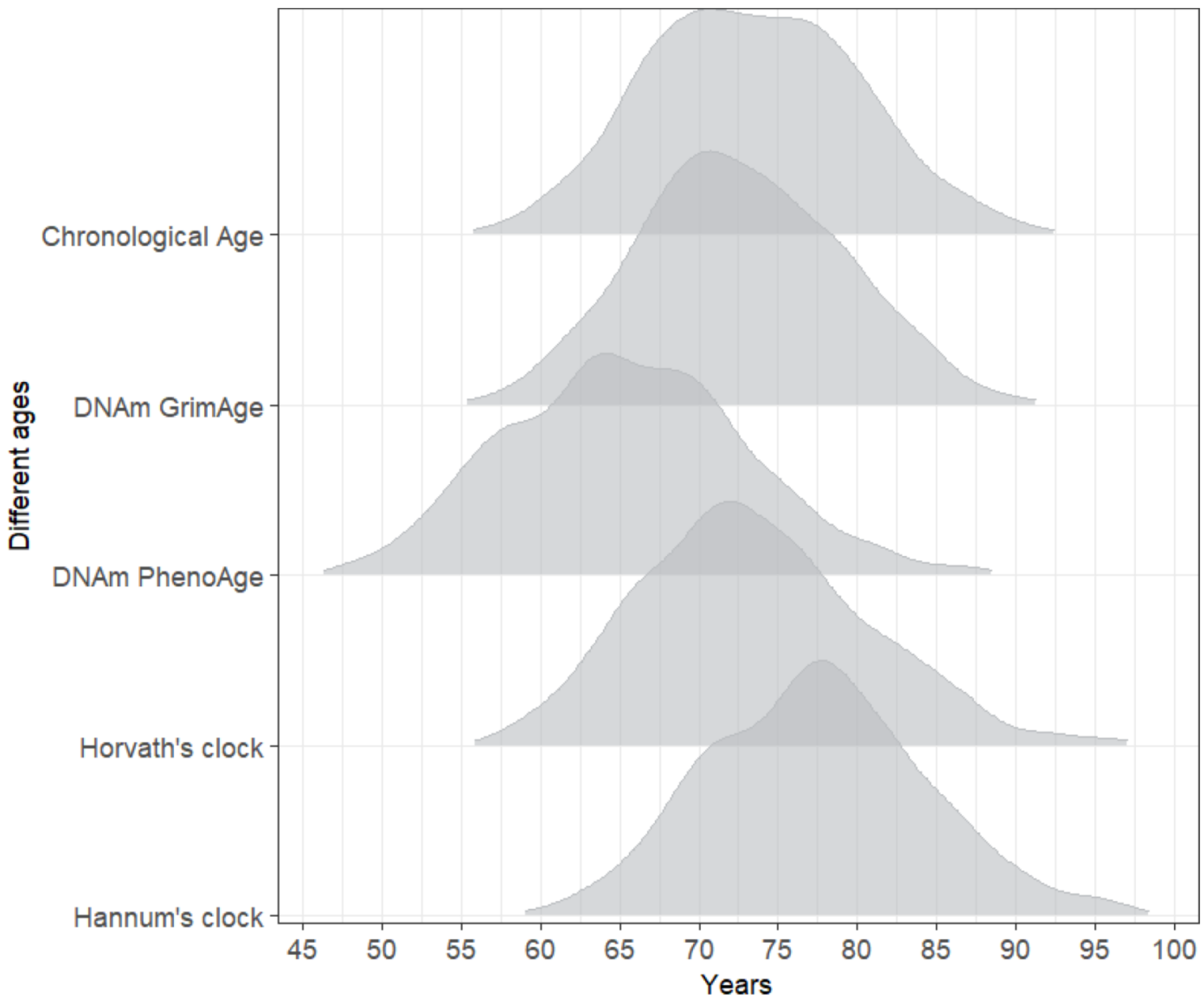

Supplementary Figure 4. Distribution of chronological age and four epigenetic ages.

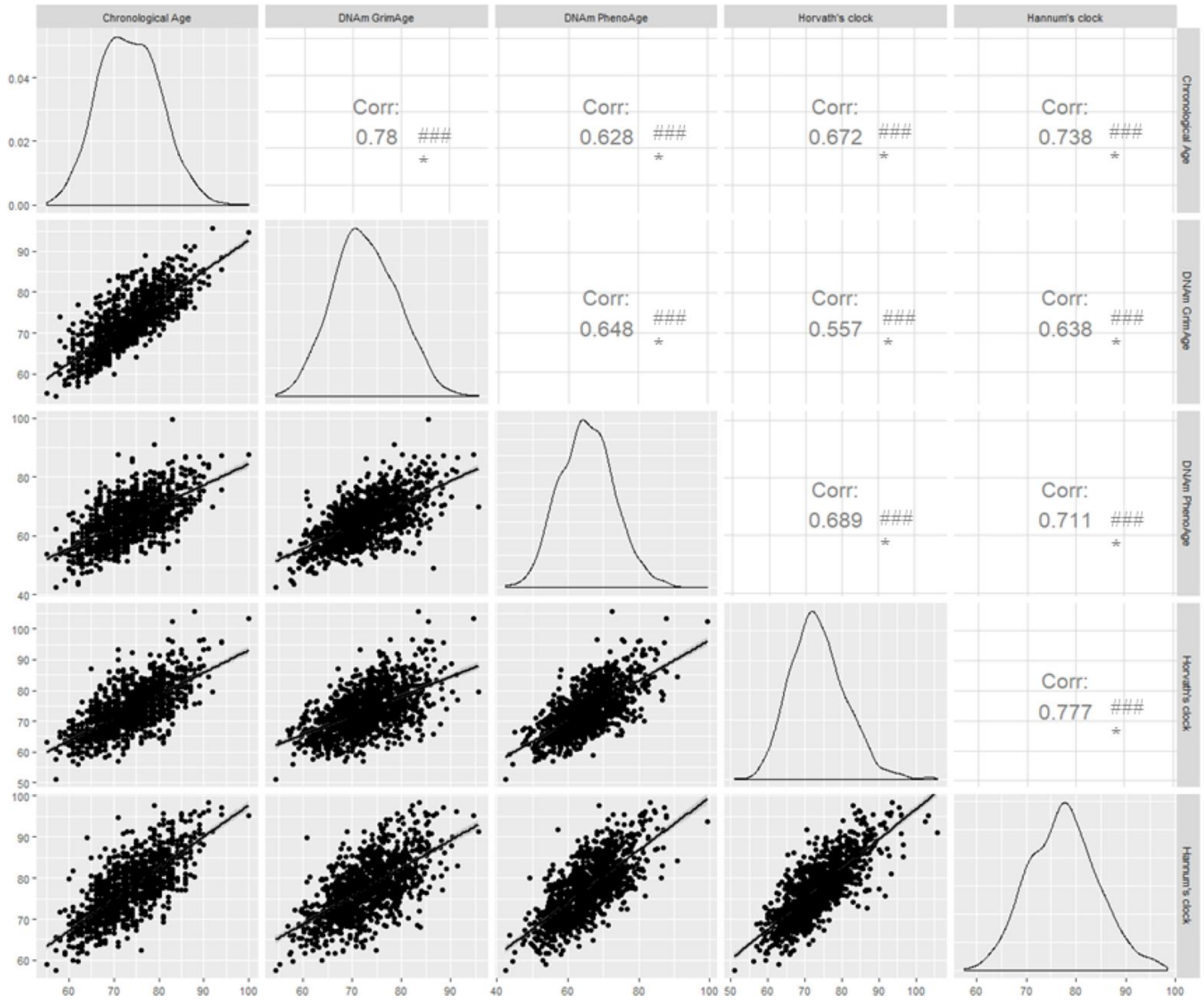

Supplementary Figure 5. Pairwise correlations between chronological age and four epigenetic ages. ### P ≤ 0.001; * Clinically significant; Corr = correlation coefficient.

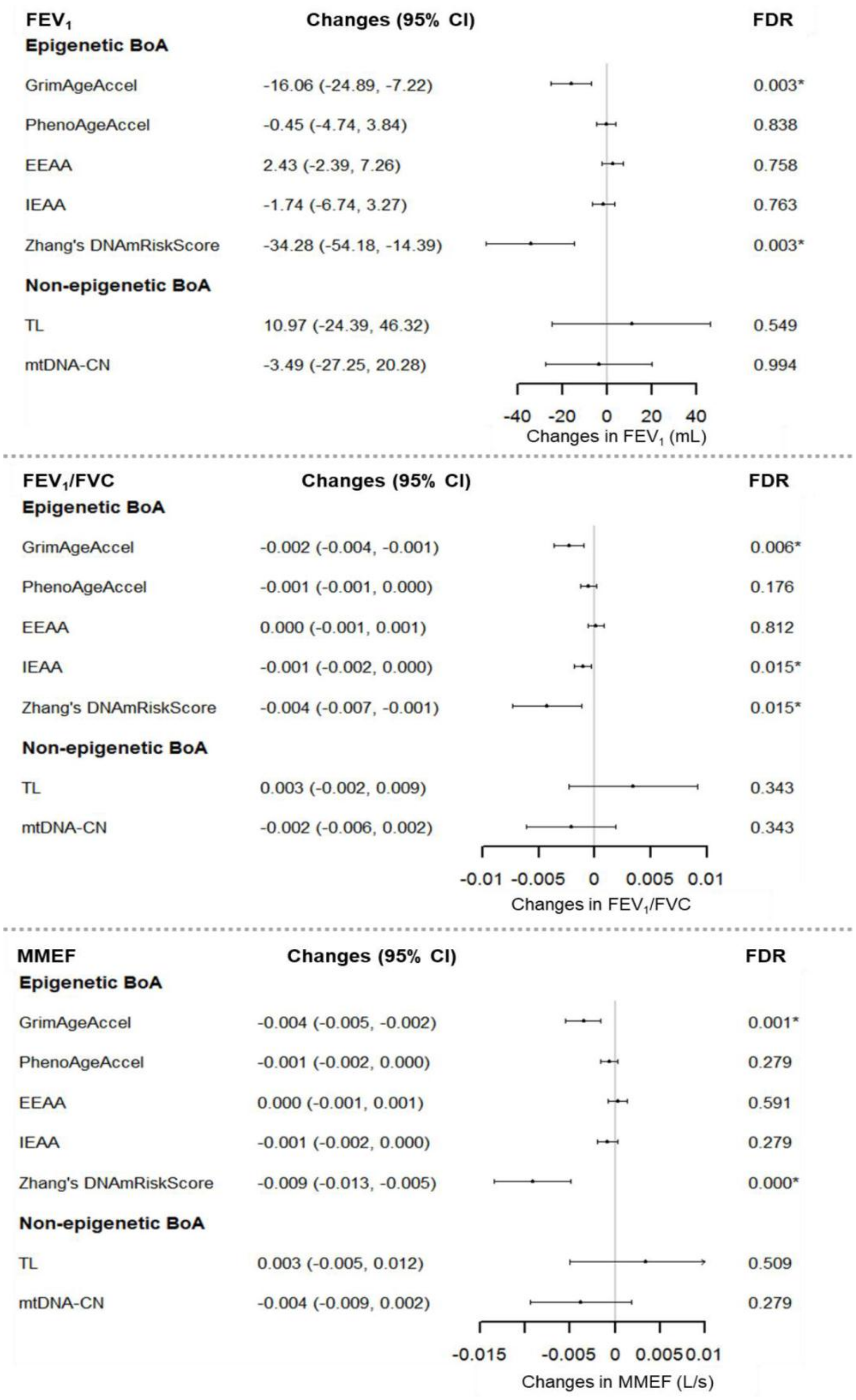

Supplementary Figure 6. Sensitivity analyses: Associations between seven BoAs and lung function after removing visits at which corticosteroid use was reported, the Normative Aging Study, 1999-2013. Abbreviations: IEAA = intrinsic epigenetic age acceleration; EEAA = extrinsic epigenetic age acceleration; TL = Telomere length; mtDNA-CN = mitochondrial DNA copy number; BoA = biomarkers of aging; BMI = body mass index; FDRB-H = Benjamin-Hochberg false discovery rate

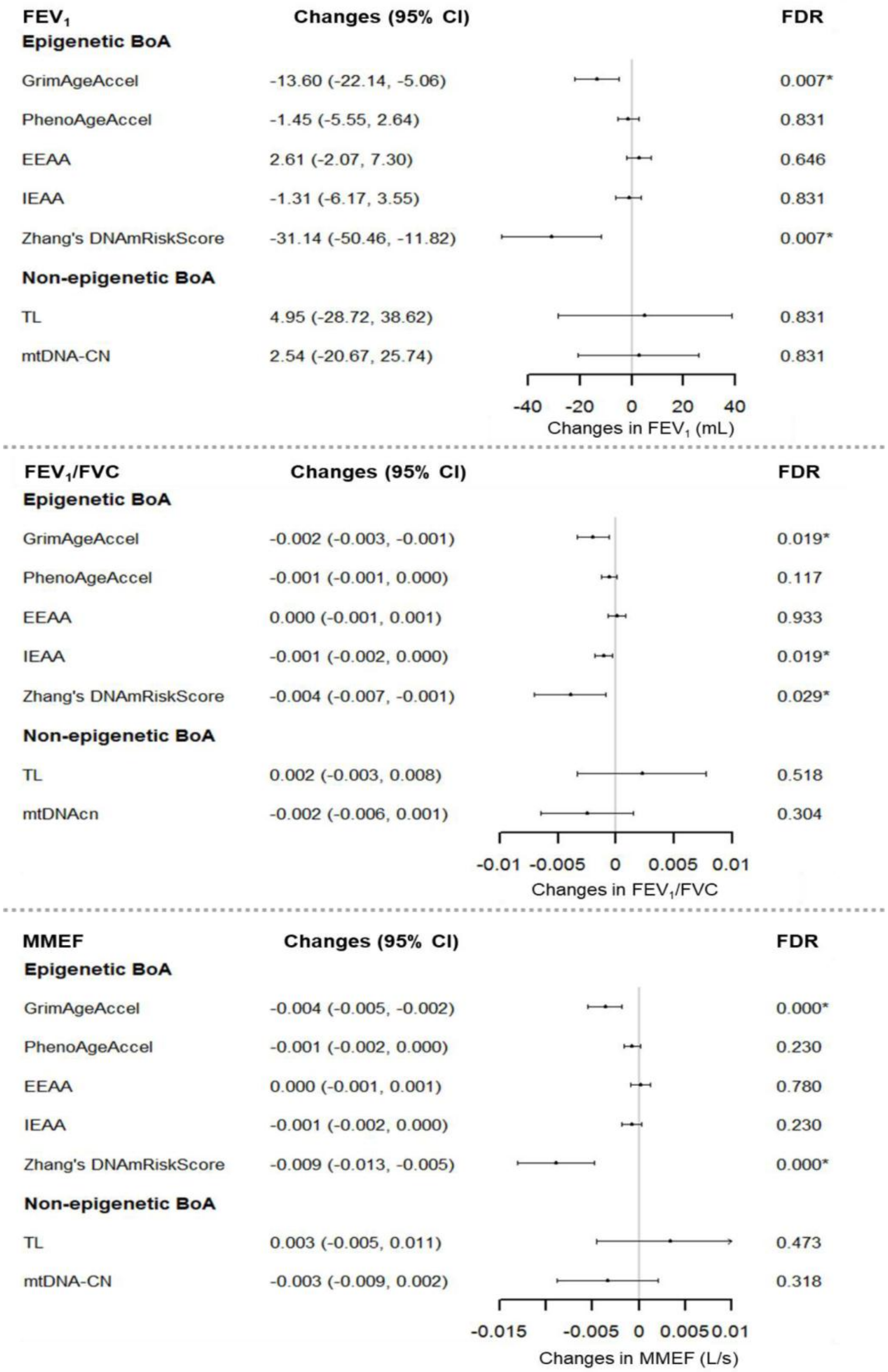

Supplementary Figure 7. Sensitivity analyses: Associations between seven BoAs and lung function additionally adjusting for coronary heart disease, stroke, and diabetes, the Normative Aging Study, 1999-2013. Abbreviations: IEAA = intrinsic epigenetic age acceleration; EEAA = extrinsic epigenetic age acceleration; TL = Telomere length; mtDNA-CN = mitochondrial DNA copy number; BoA = biomarkers of aging; BMI = body mass index; FDRB-H = Benjamin-Hochberg false discovery rate.

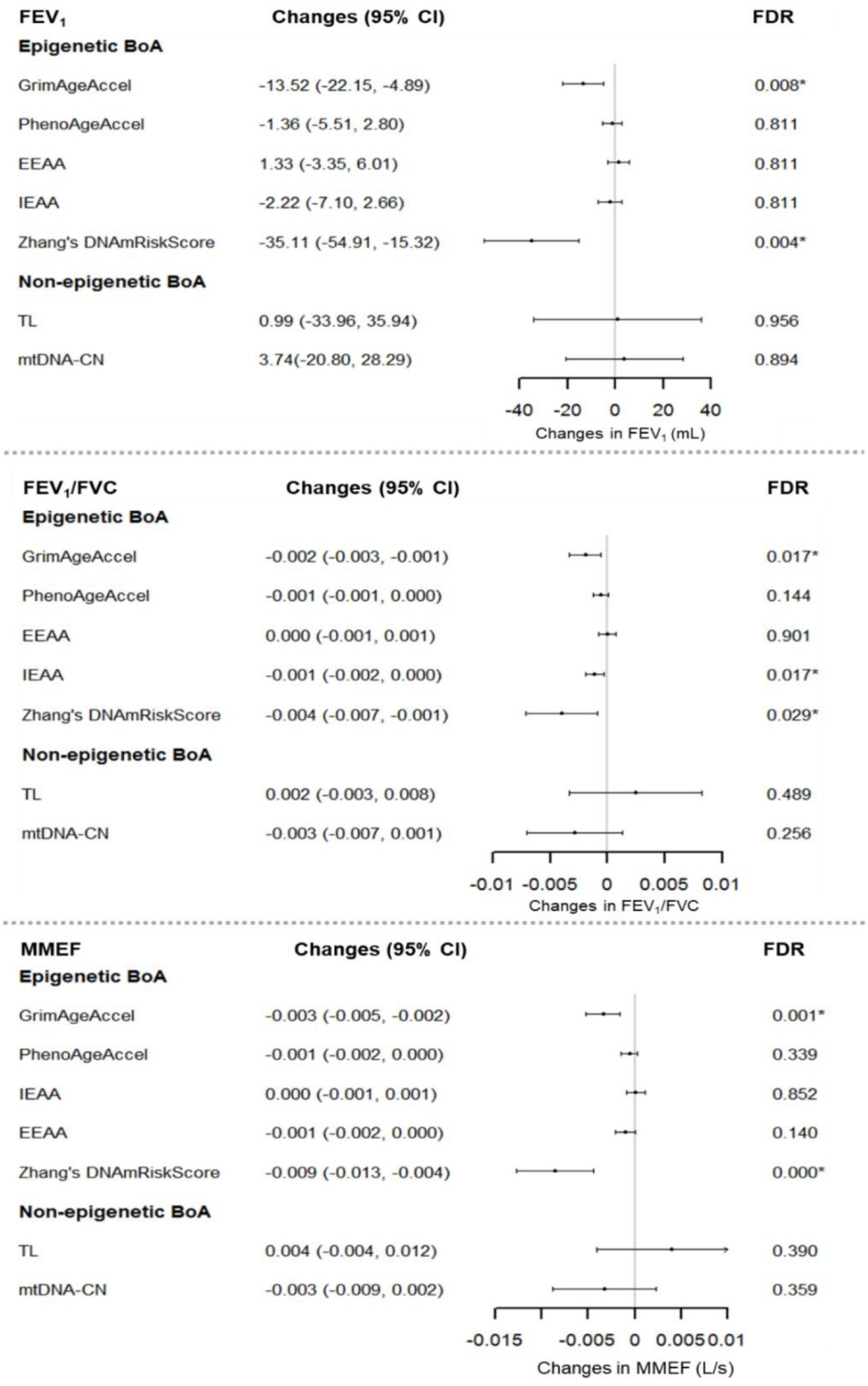

Supplementary Figure 8. Sensitivity analyses: Associations between seven BoAs and lung function additionally accounting for potential selection bias due to follow-up loss, the Normative Aging Study, 1999-2013. Abbreviation: IEAA = intrinsic epigenetic age acceleration; EEAA = extrinsic epigenetic age acceleration; TL = Telomere length; mtDNA-CN = mitochondrial DNA copy number; BoA = biomarkers of aging; BMI = body mass index; FDRB-H = Benjamin-Hochberg false discovery rate.

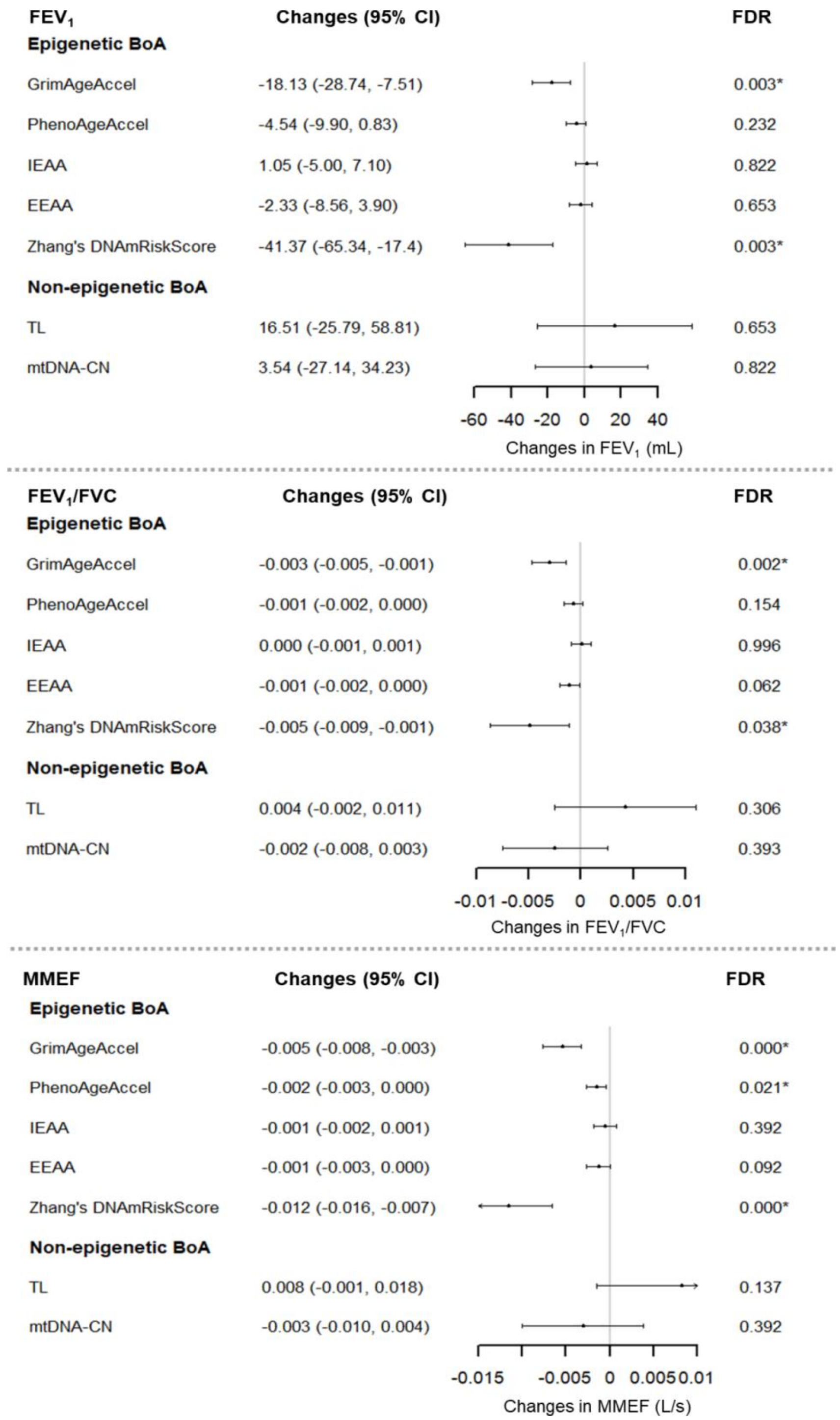

Supplementary Figure 9A. Sensitivity analyses: Associations between seven BoAs and lung function for ever smokers (729 visits, 481 men), the Normative Aging Study, 1999-2013. Abbreviations: IEAA = intrinsic epigenetic age acceleration; EEAA = extrinsic epigenetic age acceleration; TL = Telomere length; mtDNA-CN = mitochondrial DNA copy number; BoA = biomarkers of aging; BMI = body mass index; FDRB-H = Benjamin-Hochberg false discovery rate.

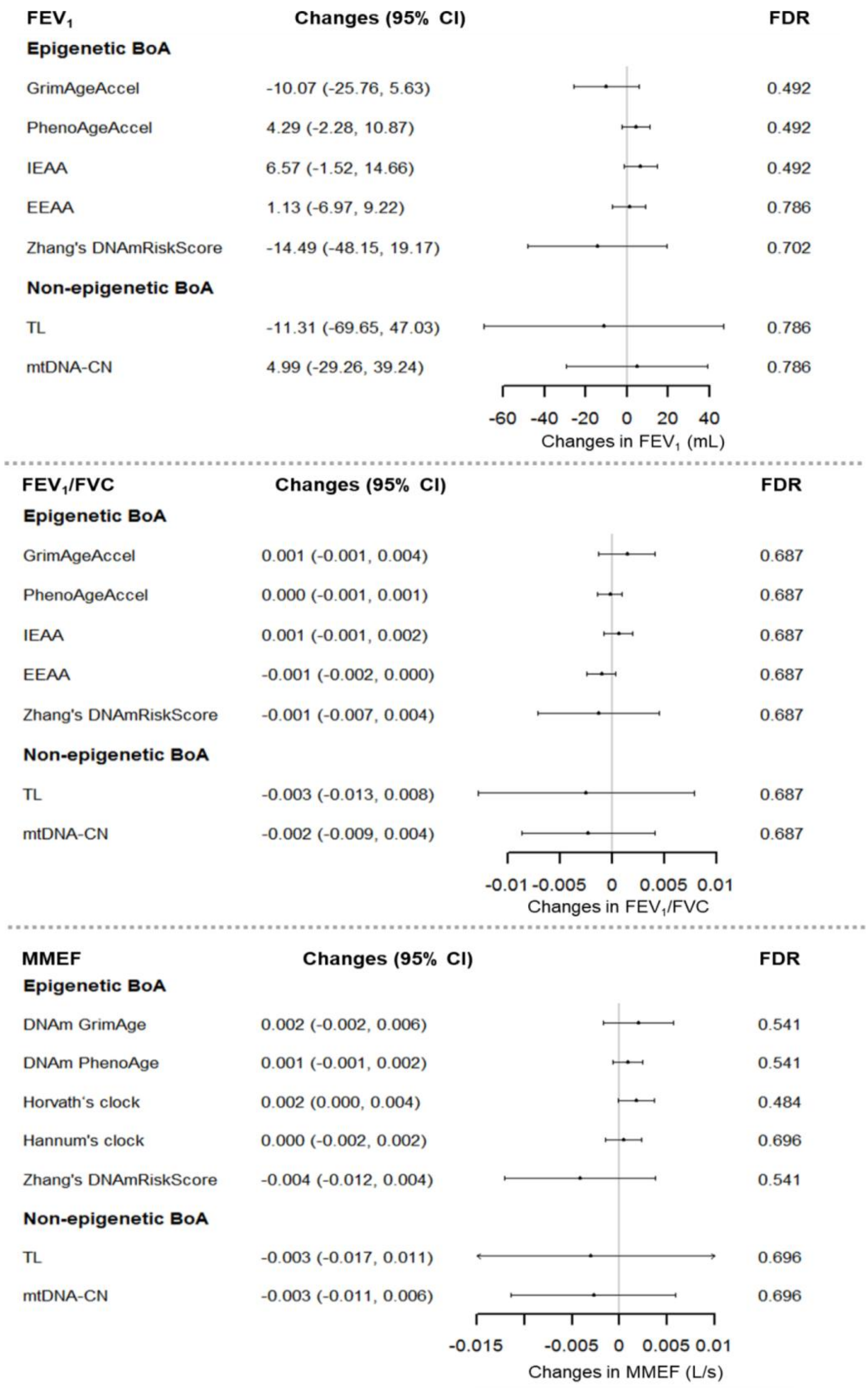

Supplementary Figure 9B. Sensitivity analyses: Associations between seven BoAs and lung function for never smokers (341 visits, 215 men), the Normative Aging Study, 1999-2013. Abbreviations: IEAA = intrinsic epigenetic age acceleration; EEAA = extrinsic epigenetic age acceleration; TL = Telomere length; mtDNA-CN = mitochondrial DNA copy number; BoA = biomarkers of aging; BMI = body mass index; FDRB-H = Benjamin-Hochberg false discovery rate.
