# Supplementary material for: Biomarkers of aging and lung function in the normative aging study
Source: Aging (Albany NY). 2020 Jun 19;12(12):11942–66. doi: 10.18632/aging.103363 (PMC7343502; doi:10.18632/aging.103363)
Supplement: Supplementary Table [file aging-12-103363-s001..pdf]

SUPPLEMENTARY TABLE

Supplementary Table 1. Summary statistics of chronological age and different DNAm ages in 1, 070 visits among 696 elderly white men from the Normative Aging Study, 1999-2013.

Variable	mean ± SD	Percentile		
		5th	50th	95th
Chronological Age (years)	73.48 ± 6.74	63.00	73.00	85.00
Epigenetic aging				
DNAm GrimAge (years)	72.72 ± 6.53	62.25	72.27	83.74
DNAm PhenoAge (years)	65.45 ± 7.72	53.24	65.21	78.13
Horvath's clock (years)	73.57 ± 7.40	62.54	72.99	86.43
Hannum's clock (years)	77.47 ± 6.97	66.54	77.44	89.24

Abbreviations: SD = standard deviation; TL = Telomere length; mtDNA-CN = mitochondrial DNA copy number.
